# Cellular Composition and 5hmC Signature Predict the Treatment Response of AML Patients to Azacitidine Combined with Chemotherapy

**DOI:** 10.1002/advs.202300445

**Published:** 2023-06-04

**Authors:** Guanghao Liang, Linchen Wang, Qiancheng You, Kirk Cahill, Chuanyuan Chen, Wei Zhang, Noreen Fulton, Wendy Stock, Olatoyosi Odenike, Chuan He, Dali Han

**Affiliations:** ^1^ Key Laboratory of Genomic and Precision Medicine Beijing Institute of Genomics Chinese Academy of Sciences and China National Center for Bioinformation Beijing 100101 China; ^2^ College of Future Technology Sino‐Danish College University of Chinese Academy of Sciences Beijing 100049 China; ^3^ Department of Chemistry and Institute for Biophysical Dynamics The University of Chicago Chicago IL 60637 USA; ^4^ Howard Hughes Medical Institute Chicago IL 60637 USA; ^5^ Section of Hematology/Oncology Department of Medicine University of Chicago Medicine Chicago IL 60637 USA; ^6^ Department of Medicine University of California, San Diego La Jolla CA 92093 USA; ^7^ Bristol‐Myers Squibb San Diego CA 92121 USA; ^8^ Comprehensive Cancer Center University of Chicago Medicine Chicago IL 60637 USA; ^9^ Department of Biochemistry and Molecular Biology The University of Chicago Chicago IL 60637 USA; ^10^ Institute for Stem Cell and Regeneration Chinese Academy of Sciences Beijing 100101 China

**Keywords:** 5hmC, acute myeloid leukemia (AML), azacitidine, biomarkers, machine learning

## Abstract

Azacitidine (AZA) is a DNA methyltransferase inhibitor and epigenetic modulator that can be an effective agent in combination with chemotherapy for patients with high‐risk acute myeloid leukemia (AML). However, biological factors driving the therapeutic response of such hypomethylating agent (HMA)‐based therapies remain unknown. Herein, the transcriptome and/or genome‐wide 5‐hydroxymethylcytosine (5hmC) is characterized for 41 patients with high‐risk AML from a phase 1 clinical trial treated with AZA epigenetic priming followed by high‐dose cytarabine and mitoxantrone (AZA‐HiDAC‐Mito). Digital cytometry reveals that responders have elevated Granulocyte‐macrophage‐progenitor‐like (GMP‐like) malignant cells displaying an active cell cycle program. Moreover, the enrichment of natural killer (NK) cells predicts a favorable outcome in patients receiving AZA‐HiDAC‐Mito therapy or other AZA‐based therapies. Comparing 5hmC profiles before and after five‐day treatment of AZA shows that AZA exposure induces dose‐dependent 5hmC changes, in which the magnitude correlates with overall survival (*p* = 0.015). An extreme gradient boosting (XGBoost) machine learning model is developed to predict the treatment response based on 5hmC levels of 11 genes, achieving an area under the curve (AUC) of 0.860. These results suggest that cellular composition markedly impacts the treatment response, and showcase the prospect of 5hmC signatures in predicting the outcomes of HMA‐based therapies in AML.

## Graphical abstract

Azacitidine (AZA) is a DNA methyltransferase inhibitor that can combine with chemotherapy for treating patients with acute myeloid leukemia. The authors show that drug response to AZA combined with chemotherapy is driven by a subset of granulocyte‐macrophage‐progenitor‐like (GMP‐like) malignant cells marked by cell‐cycle gene expression, and increased level of NK cells. Utilizing machine learning, they establish an 11‐gene 5hmC model to predict outcomes.

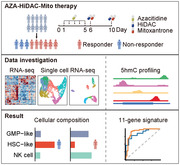

## 1 Introduction

Acute myeloid leukemia (AML) is an aggressive malignancy characterized by a low cure rate and 5‐year survival of 30–35%. The significant genetic and cellular heterogeneity of AML contributes to highly variable responses to treatment.^[^ ^1^ ^]^ Given that epigenetic aberrations arecommonly observed and implicated in the pathogenesis of AML,^[^ ^2^ ^]^ there has been an interest in combining hypomethylating agents (HMAs), azacitidine (AZA), and decitabine, with cytotoxic chemotherapy, targeted therapy, or immunotherapy with the goal to improve outcomes for patients with AML.^[^ ^3^ ^]^ While HMAs can reactivate aberrantly silenced genes, induce antiviral innate immune responses, and sensitize malignant cells to cytotoxic agents, the mechanism of their anti‐leukemic effect is not fully understood.^[^ ^4^ ^]^ Recent studies have highlighted the importance of malignant cell composition and immune landscape in determining the clinical outcomes of AML.^[^ ^1^, ^5^ ^]^ However, the exact subsets of malignant cells and immune cells that determine the therapeutic response to these HMAs are unclear.

We previously reported a phase 1 clinical trial of AZA treatment followed by high‐dose cytarabine and mitoxantrone (AZA‐HiDAC‐Mito) in high‐risk AML patients, based on the hypothesis that epigenetic priming with a HMA (AZA) would sensitize malignant cells to cytotoxic therapy.^[^ ^6^ ^]^ The overall response rate [(complete remission (CR) + CR with incomplete count recovery (CRi)] in this phase 1 study was 61% with a low induction death rate of 2.2%. While AZA‐HiDAC‐Mito trended toward a higher response rate compared to a historical cohort treated with HiDAC‐Mito alone,^[^ ^6^, ^7^ ^]^ the pre‐treatment determinants and biomarkers for a treatment strategy including epigenetic priming remain unknown.

Although gene expression and epigenetic profiling have yet to be adopted routinely in clinical practice, such approaches may help with prognostication and treatment decisions in AML.^[^ ^8^ ^]^ Cytosine methylation (5mC) is a well‐established epigenetic biomarker involved in cancer development and progression.^[^ ^9^ ^]^ 5mC is maintained by DNA methyltransferases, while the TET family of dioxygenases convert 5mC to 5‐hydroxymethylcytosine (5hmC) in an active demethylation process.^[^ ^10^ ^]^ Increasing evidence suggests that 5hmC levels are related to tumorigenesis, including observations that global 5hmC levels are reduced in various cancer types.^[^ ^2^, ^11^ ^]^ Furthermore, recent studies have demonstrated that AZA treatment affects the cellular level and genomic distribution of 5hmC,^[^ ^12^ ^]^ which could be used as a robust diagnostic and prognostic biomarker for broad cancer types.^[^ ^13^ ^]^ These studies support 5hmC as an ideal candidate for an epigenetic biomarker to predict the outcomes of AZA‐HiDAC‐Mito therapy.

Herein, to elucidate the underlying mechanisms of treatment response for AZA‐HiDAC‐Mito therapy, we collected samples in a phase 1 clinical study and performed RNA‐seq and 5hmC profiling. By combining the public single‐cell RNA‐seq data, we found that responders highly expressed cell‐cycle‐related genes, which were inferred to be expressed primarily by a subset of Granulocyte‐macrophage‐progenitor‐like (GMP‐like) malignant cells. In contrast, hematopoietic stem cell‐like (HSC‐like) malignant cells with low expression of cell‐cycle‐related genes were more likely to be enriched in non‐responders. Moreover, we found that AZA treatment induced gene expression related to NK cell cytotoxicity in responders. In line with this, the pre‐treatment level of NK cells was associated with improved clinical outcome of AZA‐HiDAC‐Mito therapy. By analyzing samples from patients receiving AZA treatment, compared to those receiving decitabine treatment or standard chemotherapy in the Beat AML cohort, and our historical HiDAC‐Mito cohort, we demonstrated the specific role of NK cells in response to AZA‐based treatment. Furthermore, AZA exposure induced a dose‐dependent alteration in 5hmC after treatment for five days, and patients with more pronounced changes in 5hmC modifications exhibited improved survival. We then developed a machine learning prediction model based on 5hmC levels in 11 genes, which accurately predicted treatment response.

## 2 Results

### 2.1 Activation of the Cell Cycle Program was Associated with Response to AZA‐HiDAC‐Mito Therapy

**Figure 1:**
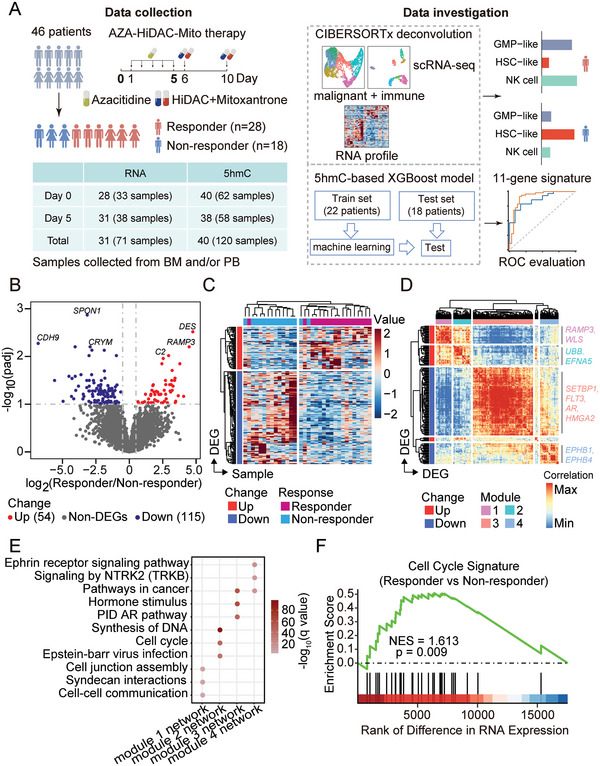
Differential expression analysis between responders and non‐responders of AZA‐HiDAC‐Mito therapy. A) Diagram of study design, therapeutic strategy, and data analysis workflow. RNA and DNA obtained from peripheral blood and bone marrow samples of 46 AML patients receiving AZA‐HiDAC‐Mito therapy were used in this study (41 patients provided usable DNA/RNA samples). The figure in the table is the number of patients. B) Volcano plot showing gene expression difference between responders and non‐responders. Thirty‐three BM and/or PB samples obtained from 28 patients were used. *p* values were calculated with the Wald test and adjusted by the Benjamini‐Hochberg method. padj, adjusted *p* value. Top 3 DEGs for both upregulated and downregulated genes were labeled. C) Heatmap showing the expression levels of 169 DEGs in 33 AML samples collected at Day 0. Hierarchical clustering was performed across genes and samples. D) Heatmap showing hierarchical clustering of the pairwise correlations among DEGs in 33 AML samples. DEGs were grouped into four major modules. E) Functional enrichment for genes in each module‐related network. The module‐related network was obtained from the STRING database by adding the directly interacting genes of the DEGs. The *q* value was adjusted *p* value by the Benjamini‐Hochberg method. F) GSEA to assess the enrichment of cell cycle signature in responders of AZA‐HiDAC‐Mito therapy. NES, normalized enrichment score; *p* value was calculated with permutation test.

A total of 46 patients who received AZA‐HiDAC‐Mito therapy were enrolled in this study, out of which 41 provided usable RNA and/or 5hmC sequencing data (**Figure** **1**A; Table S1, Supporting Information). Of these patients, 19/46 (41%) achieved complete remission (CR) after treatment, with 9/46 (20%) diagnosed as CR but with incomplete count recovery (CRi), and 18/46 (39%) experienced treatment failure (TF). The overall response rate was 61% (28/46). The results of this trial have been previously published.^[^ ^6^ ^]^

To identify gene expression programs that may confer sensitivity to AZA‐HiDAC‐Mito treatment, we collected mononuclear cells from bone marrow (BM) and/or peripheral blood (PB) prior to AZA treatment (Day 0) and performed RNA‐seq. Twenty‐eight patients had pre‐treatment material available for RNA‐seq. By comparing the gene expression levels between responders (CR + CRi, *n* = 16) and non‐responders (*n* = 12), we identified 54 upregulated and 115 downregulated genes in responders (Figure 1B). Unsupervised clustering analysis revealed that these differentially expressed genes (DEGs) were capable of distinguishing responders from non‐responders (Figure 1C), suggesting that the transcriptional profile was tightly associated with treatment response.

To shed light on the potential mechanism(s) of treatment response, we performed an extended co‐expression network analysis by integrating protein association networks from the STRING (Search Tool for the Retrieval of Interacting Genes/Proteins) database.^[^ ^14^ ^]^ Briefly, we first identified four gene modules through co‐expression analysis, including *RAMP3* (signaling receptor activity), *EFNA5* (G2M checkpoint), *FLT3* (hematopoiesis), *EPHB1* (ephrin receptor) for module 1–4, respectively (Figure 1D). Genes within the same co‐expression module are highly correlated and probably have similar biological functions. Next, we extended each gene module by adding first‐order neighbors in the STRING database to construct a functional network. Enrichment analysis revealed that genes in modules 3 and 4, which were downregulated in responders, are enriched for pathways known to be involved in tumorigenesis and tumor progression, such as ephrin receptor signaling pathway (Figure 1E).^[^ ^15^ ^]^ In contrast, for the two modules that were upregulated in responders, we observed that the functional network for module 1 is enriched for cell‐cell interaction and communication, while genes within the module 2 network are involved in cell cycle and DNA synthesis.

Since both AZA and cytarabine are known to interfere with DNA synthesis and preferentially eliminate cycling cells,^[^ ^16^ ^]^ we reasoned that activation of the cell cycle program may be associated with response to AZA‐HiDAC‐Mito therapy. To this end, we performed gene set enrichment analysis (GSEA) to evaluate the enrichment of a curated cell cycle signature in responders compared to non‐responders.^[^ ^17^ ^]^ We found that expression of the cell cycle signature was highly enriched in responders, which suggests that patients with activated cell cycle program are sensitive to AZA‐HiDAC‐Mito therapy (Figure 1F).

### 2.2 Elevated GMP‐Like Malignant Cells with Active Cell Cycle Program Predicted Treatment Response

**Figure 2:**
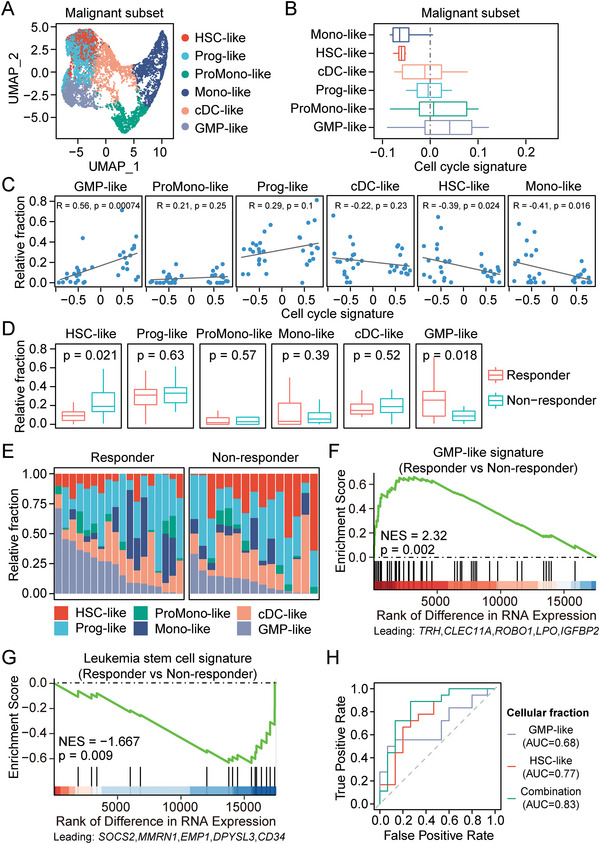
AML malignant composition correlated with treatment response. A) UMAP visualization of malignant subsets from the public single‐cell transcriptome (van Galen et al). Six subsets of malignant cells were included: hematopoietic stem cell‐like (HSC‐like), progenitor‐like (Prog‐like), promonocyte‐like (ProMono‐like), monocyte‐like (Mono‐like), conventional dendritic cell‐like (cDC‐like), Granulocyte‐macrophage‐progenitor‐like (GMP‐like). B) Boxplot showing the aggregated gene expression of cell cycle signature in each malignant subset per patients in the scRNA‐seq dataset. C) Pearson's correlation between the estimated abundance of malignant subsets and the overall expression of cell cycle signature in pre‐treatment RNA‐seq samples. D) Boxplot showing the estimated relative abundance of each malignant subset. *p* values were calculated with two‐sided Student's *t*‐test. Fractions of BM/PB samples from same patients were averaged. E) The relative abundance of each malignant subset in each pre‐treatment sample. F,G) GSEA to assess the enrichment of GMP‐like signature F) and LSC17 signature G) in responders of AZA‐HiDAC‐Mito therapy, comparing to non‐responders. NES, normalized enrichment score; *p* value was calculated with permutation test. H) Receiving operating curve (ROC) analysis: Using relative fraction of GMP‐like cells, HSC‐like cells, or their combination (difference between the fractions of GMP‐like cells and HSC‐like cells) to predict responders.

The cycling status of AML malignant cells is known to be heterogeneous.^[^ ^16^, ^18^ ^]^ We next sought to determine the malignant subsets that are in active cell cycle and likely sensitive to AZA‐HiDAC‐Mito therapy. By analyzing the public single‐cell RNA sequencing (scRNA‐seq) profiles of AML samples from 12 patients,^[^ ^19^ ^]^ we compared six distinct subsets of malignant cells and seven immune cell types (**Figure** **2**A; Figure S1A, Supporting Information). As expected, cell cycle signature was prominently expressed in several malignant subsets (Figure 2B; Figure S1B, Supporting Information). Notably, GMP‐like cells exhibited the highest expression of cell cycle signature among the malignant subsets, while HSC‐like and monocyte‐like (Mono‐like) malignant cells exhibited the lowest expression of cell cycle signature. Next, we applied the digital cytometry method, CIBERSORTx, to deconvolute the pre‐treatment RNA‐seq samples and estimate the abundance of each cell type based on the single‐cell reference profiles (Figure S1C, Supporting Information).^[^ ^20^ ^]^ We observed a positive correlation between the estimated abundance of GMP‐like cells and the overall expression of the cell cycle signature in bulk RNA‐seq samples, whereas there were negative correlations for both HSC‐like and Mono‐like cells (Figure 2C). It is noteworthy that the HSC‐like malignant cells were highly similar to previously defined leukemia stem cells (LSCs), which are known to be in a quiescent and non‐dividing state (Figure S1D, Supporting Information).^[^ ^21^ ^]^ These results indicate that the global cycling status of AML malignant cells is closely related to the malignant cell composition.

Next, we questioned whether the compositions of malignant subsets were linked to treatment responses to AZA‐HiDAC‐Mito therapy. Remarkably, GMP‐like cells were more abundant in responders, while HSC‐like cells were enriched in non‐responders (Figure 2D,E; Figure S1E, Supporting Information). This observation was further supported by the results of GSEA enrichment analysis conducted on the previously reported GMP‐like signature and LSC17 gene signature (Figure 2F,G).^[^ ^19^, ^21^ ^]^ We also employed Gene Set Variation Analysis (GSVA) to establish a GMP score based on the GMP‐like signature for each sample, and compared it with the well‐established LSC17 score.^[^ ^21a^ ^]^ Both signatures exhibited AUC = 0.71 in distinguishing responders and non‐responders (Figure S1F, Supporting Information). Similar performance was observed when using the relative fraction of GMP‐like cells or HSC‐likes cells as an indicator (AUC = 0.68 and 0.77, respectively). Furthermore, combining these two cellular fractions using their difference resulted in superior performance with an AUC value of 0.83 (Figure 2H). Specifically, a malignant composition that is GMP‐like‐dominant predicts treatment response, while an HSC‐like‐dominant malignant composition is associated with treatment failure (Figure S1G, Supporting Information). Taken together, our results highlight GMP‐like cells as the primary malignant subset that is sensitive to AZA‐HiDAC‐Mito therapy owing to an active cell cycle program, and the difference in cellular fractions between GMP‐like and HSC‐like malignant subsets can serve as a predictive indicator of responders to AZA‐HiDAC‐Mito therapy.

### 2.3 AZA Treatment Induced Upregulation of Genes Related to Natural Killer Cell Mediated Cytotoxicity in Responders

**Figure 3:**
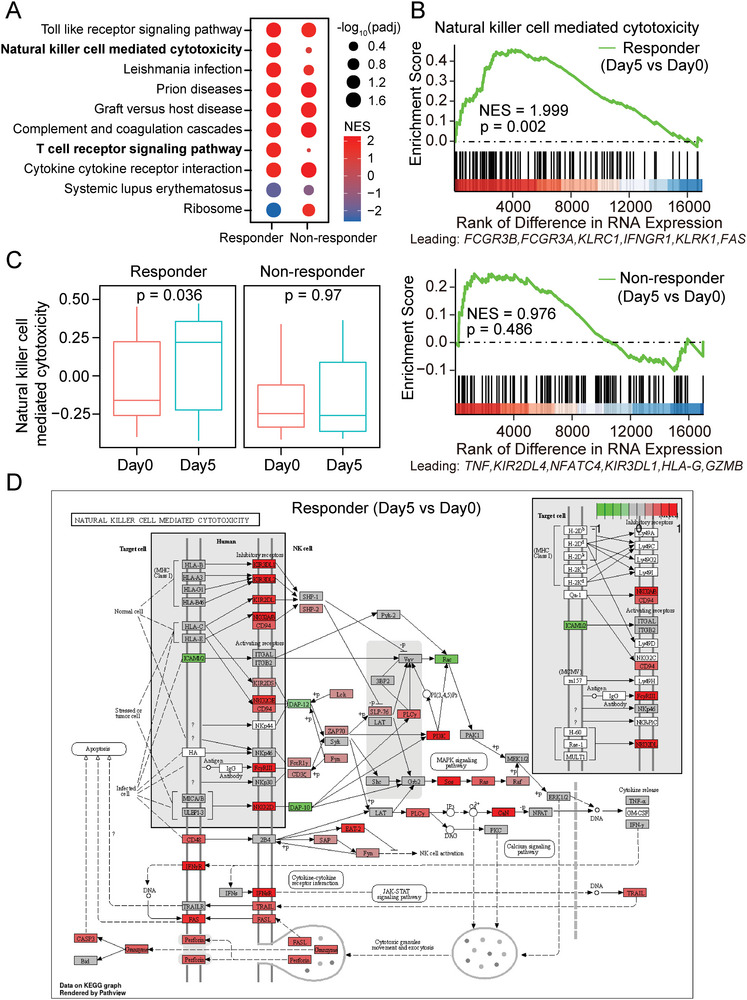
Transcriptional changes upon AZA treatment for 5 days. A) Bubble plot showing the results of GSEA analysis on KEGG pathways by comparing gene expression between Day 5 and Day 0. NES, normalized enrichment score. Top 10 enriched pathways in responders are shown. padj, adjusted *p* value. NES, normalized enrichment score. B) GSEA to assess the enrichment of Natural killer cell mediated cytotoxicity pathway upon AZA treatment for 5 days in responders (top) and non‐responders (bottom). NES, normalized enrichment score; *p* value was calculated with permutation test. C) Boxplot showing GSVA scores of Natural killer cell mediated cytotoxicity pathway. Paired samples were used in comparison of Day 5 and Day 0. *p* values were calculated with two‐sided paired Student's *t*‐test. D) Pathview map showing the gene expression changes between Day 5 and Day 0 in responders in Natural killer cell mediated cytotoxicity pathway. The mapped color indicates log_2_(fold change) of Day 5 versus Day 0.

Much effort had been made to identify the transcriptional effects upon epigenetic priming by AZA treatment in both solid tumors and hematologic malignancies, providing insight into the mechanisms by which AZA treatment exerts its effects.^[^ ^4^, ^22^ ^]^ Nevertheless, the in vivo transcriptional effects of AZA treatment in AML and their association to clinical response to an AZA‐based therapy are still unclear. To address this, we compared gene expression levels between RNA‐seq samples from Day 5 and Day 0. GSEA analysis on KEGG (Kyoto Encyclopedia of Genes and Genomes) pathways revealed that AZA treatment induced upregulation of multiple pathways related to immune processes and immune activation (**Figure** **3**A). Specifically, natural killer cell mediated cytotoxicity and T cell receptor signaling pathways were only upregulated in responders, suggesting distinct effects upon AZA treatment between responders and non‐responders (Figure 3B; Figure S2A, Supporting Information). We further calculated gene‐set enrichment scores per sample with GSVA, and observed the pairwise upregulation of natural killer cell mediated cytotoxicity pathway but not T cell receptor signaling pathway in responders (Figure 3C; Figure S2B, Supporting Information). We then mapped the transcriptional changes onto the KEGG pathway using Pathview,^[^ ^23^ ^]^ and observed a global upregulation of components in natural killer cell mediated cytotoxicity pathway in responders (Figure 3D; Figure S2C, Supporting Information).

### 2.4 Enrichment of NK Cells Predicted Favorable Clinical Outcomes in AZA‐Based Therapies

**Figure 4:**
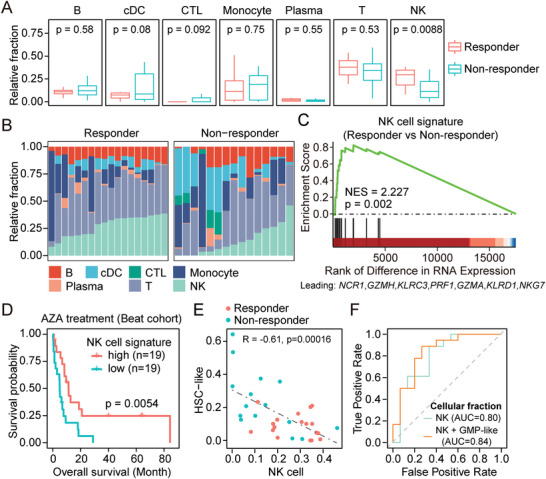
Immune landscape correlated with treatment response. A) Boxplot showing the estimated relative abundance of each immune subset. *p* values were calculated with two‐sided Student's *t*‐test. Fractions of BM/PB samples from same patients were averaged. B) The relative abundance of each immune population in each sample from patients prior to AZA treatment. C) GSEA to assess the enrichment of a curated NK cell signature in responders of AZA‐HiDAC‐Mito therapy, comparing to non‐responders. NES, normalized enrichment score; *p* value was calculated with permutation test. D) Kaplan‐Meier curve of overall survival for patients receiving AZA treatment in Beat AML cohort. Patients were equally divided into two groups based on the aggregated expression of a NK cell signature. *p* value was calculated with a two‐tailed log rank test. E) Pearson's correlation of cellular fractions between HSC‐like cells and NK cells in Day 0 samples. F) ROC curve for the performance of classifiers based on the cellular fraction of NK cells or the combination of NK cells and GMP‐like cells (NK + GMP‐like).

Previous studies reported that AZA treatment facilitated the tumor recognition of AML cells by NK cells.^[^ ^24^ ^]^ Our results provided in vivo evidence to support previous studies and further indicated the involvement of NK cells in determining the treatment response to such AZA‐based therapy. To test whether the baseline level of NK cells is associated with treatment response, we analyzed the deconvolution result for immune subsets in pre‐treatment RNA‐seq samples. Notably, responders had a significantly higher proportion of NK cells (*p* = 0.0088) (**Figure** **4**A,B; Figure S3A, Supporting Information), which was further supported by the enrichment of a curated NK cell signature with normalized enrichment score = 2.227, *p* = 0.002 (Figure 4C).^[^ ^25^ ^]^ The core enriched genes included NK cell receptor *NCR1* (NKp46), *KLRC3*, *KLRD1*, and NK cell cytotoxicity molecules: *GZMH*, *PRF1*, *GZMA*, and *NKG7*. These findings underlined an important role of NK cell abundance and activity in treatment response to AZA‐HiDAC‐Mito therapy.

Furthermore, we tested whether the association of NK cell abundance and treatment response is AZA‐specific. Analyzing RNA‐seq samples from a historical cohort of AML patients receiving HiDAC‐Mito only therapy revealed that neither the estimated abundance of NK cells nor the expression of NK cell signature was correlated with response to HiDAC‐Mito only therapy (Figure S3B,C, Supporting Information).^[^ ^6^ ^]^ We also assessed the expression of known NK cell marker genes *NCR1, KLRD1, NKG7*, and *KLRC3*, which were upregulated in responders of AZA‐HiDAC‐Mito therapy but not HiDAC‐Mito therapy, compared to non‐responders (Figure S3D, Supporting Information). Nevertheless, when analyzing RNA‐seq samples from patients receiving AZA treatment in Beat AML cohort,^[^ ^1c^ ^]^ we found that both upregulation of NK cell signature and elevated NK cell abundance is associated with better overall survival (Figure 4D; Figure S3E, Supporting Information). In multivariate Cox regression models, stratifying patients based on both methods retained significance for overall survival when age, sex, and ELN2017 risk classification were considered (Table S2, Supporting Information). Additionally, for patients receiving decitabine, or standard “7+3” chemotherapy (Cytarabine, Idarubicin) in the Beat AML cohort, we observed no such associations (Figure S3F,G, Supporting Information). Therefore, our results highlight the involvement of NK cells in determining the clinical outcomes of AZA‐based therapies, which may link to the in vivo effect of AZA treatment in inducing genes related to natural killer cell mediated cytotoxicity.

We next asked whether the abundance of NK cells was associated with the malignant composition. We found that the fraction of NK cells inversely correlated with the fraction of HSC‐like cells (Figure 4E; Figure S3H, Supporting Information). Therefore, the fractions of NK cells can also be combined with fractions of GMP‐like cells to classify responders and non‐responders with an AUC = 0.84, which is better than merely using the fractions of NK cells (Figure 4F). Together, our findings establish that the malignant composition and immune landscape could stratify patients with different responses to AZA‐HiDAC‐Mito therapy.

### 2.5 AZA Treatment Induced Dose‐Dependent 5hmC Changes which were Prognostic

**Figure 5:**
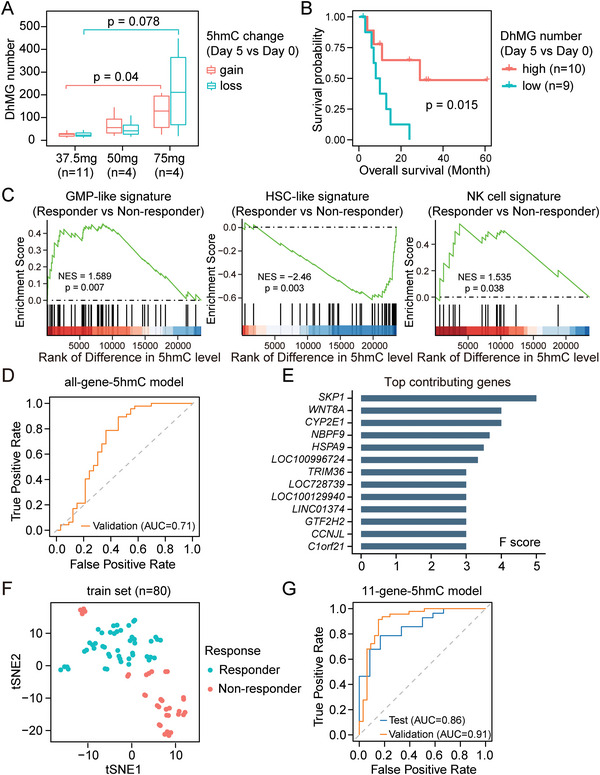
Identification of 5hmC gene signature for prediction of treatment response to AZA‐HiDAC‐Mito therapy. A) Boxplot showing the number of identified DhMGs between Day 0 and Day 5 for patients treated with different AZA doses. Nineteen patients with paired Day 0 and Day 5 samples from both BM and PB were selected (4 samples for each patient). *p* values were calculated with Wilcoxon rank sum test. B) Kaplan‐Meier survival curve for overall survival of 19 patients with paired Day 0 and Day 5 samples from both BM and PB. Patients were divided into two groups based on the median of identified DhMGs number. *p* value was calculated with a two‐tailed log rank test. C) 5hmC‐based GSEA to assess the enrichment of GMP‐like, HSC‐like, NK cell signature in responders of AZA‐HiDAC‐Mito therapy, comparing to non‐responders. NES, normalized enrichment score; *p* value was calculated with permutation test. D) ROC curve for the performance of the XGBoost classifier in cross validation for patients receiving AZA‐HiDAC‐Mito therapy. The model was trained on 80 samples. E) Bar graph showing F scores of top contributing genes in the 5hmC‐based XGBoost model. F) t‐SNE (t‐distributed stochastic neighbor embedding) plot of samples from responders and non‐responders based on 5hmC profiles of the top 142 contributing genes from the 5hmC‐based XGBoost model. G) ROC curve for the performance of the XGBoost classifier based on the 11‐gene‐5hmC signature. The model was evaluated using patient‐based five‐fold cross validation (80 samples from 22 AML patients). 40 samples from 18 AML patients were used for testing.

AZA treatment is known to affect the genome‐wide distribution of DNA 5mC and 5hmC modifications, which have been widely used for the diagnosis and prognosis of various types of cancer.^[^ ^13a–d^ ^]^ To understand the epigenetic modulation effect of AZA treatment, we characterized genome‐wide 5hmC profiles for 120 BM and/or PB samples obtained from 40 patients at Day 0 and/or Day 5 through Nano‐hmC‐Seal.^[^ ^26^ ^]^ There were only 19 patients that had both BM and PB samples at Day 0 and Day 5, and we performed differential analysis on 5hmC levels between Day 0 and Day 5 for each patient. We found that the differentially hydroxy‐methylated genes (DhMGs) between Day 0 and Day 5 were rarely shared among patients, and the intrinsic differences at 5hmC patterns between patients far outweighed the effects of AZA treatment (Figure S4A,B, Supporting Information). Nevertheless, we observed more DhMGs in patients receiving higher dose of AZA, suggesting a dose‐dependent epigenetic modulation effect of AZA treatment (**Figure** **5**A). We further used Spearman's correlation to evaluate the global difference of 5hmC profiles between Day 0 and Day 5 (29 patients with paired samples from BM were included). Indeed, a higher dose of AZA treatment led to a more discriminated 5hmC profile, reflecting a higher level of 5hmC alteration upon AZA treatment for 5 days (Figure S4C, Supporting Information).

Notably, we found that patients with higher level of 5hmC alteration upon AZA treatment for 5 days (i.e., higher number of DhMGs or lower correlation between Day 5 and Day 0) had longer overall survival in both univariate and multivariable analysis that incorporated age and sex features (Figure 5B; Figure S4D and Table S3, Supporting Information). Taken together, the epigenetic responsiveness to AZA treatment may be positively associated with patient survival, as reflected by the alteration on 5hmC modifications.

### 2.6 Predicting the Treatment Response to AZA‐HiDAC‐Mito via a 5hmC‐Based Machine Learning Model

Given that 5hmC levels are known to correlate with gene expression levels,^[^ ^27^ ^]^ we wondered whether responders and non‐responders could be also distinguished at 5hmC level in a manner similar to the RNA level. We performed differential hydroxymethylation analysis on 62 pre‐treatment 5hmC samples collected from 40 patients (Figure 1A). The extent of differences at 5hmC levels positively correlated with differences at RNA levels, especially for the DEGs (Figure S4E, Supporting Information). Additionally, we evaluated the enrichment of gene signatures for GMP‐like cells, HSC‐like cells, and NK cells with GSEA analysis based on 5hmC levels, which exhibited consistent enrichment patterns similar to RNA levels (Figure 5C). These data suggest that 5hmC can also be used to distinguish responders and non‐responders, similar to RNA, and thus support 5hmC as a candidate biomarker for prediction of treatment response.

In comparison to RNA‐based biomarkers, DNA‐based biomaterials are far more stable during collection, handling, and transportation. As implicated in tumorigenesis and disease progression, DNA 5hmC modifications have been widely used for the diagnosis and prognosis of various types of cancer.^[^ ^13^ ^]^ We thus tried to identify a 5hmC gene signature for prediction of treatment response by employing the eXtreme Gradient Boosting (XGBoost) machine learning algorithm to build a classifier model. Since the 5hmC profiles from samples collected at Day 0 and Day 5 were highly analogous for the same patient at genome‐wide level (Figure S4B, Supporting Information), we included both Day 0 and Day 5 samples to enlarge the sample size. The 5hmC profiles were divided into a train set and test set based on sequencing batches (80 samples from 22 patients sequenced in the first batch were used as the train set; 40 samples from 18 patients sequenced in the second batch were used as the test set). We initially trained an XGBoost model with all genes on the train set and evaluated its performance with patient‐based five‐fold cross validation.

The receiver operating characteristic (ROC) curve showed that the 5hmC XGBoost classifier achieved AUC = 0.71 in cross validation (Figure 5D; Figure S4F, Supporting Information). By evaluating the F score for each gene, we identified 142 genes that contributed to the model (Figure 5E). Top contributing genes included S‐phase kinase‐associated protein 1 (*SKP1*), the component of SKP1‐CUL1‐F‐box‐protein (SCF) complex that is involved in the proteolysis of cell cycle regulators.^[^ ^28^ ^]^ Using t‐distributed stochastic neighbor embedding (t‐SNE) dimensionality reduction, the 5hmC levels of these 142 genes separate the responders and non‐responders (Figure 5F). To further obtain a 5hmC biomarker set with the best performance, we selected the genes with the highest contribution to the model to re‐build the classifier with train set samples. The signature composed from the top 11 contributing genes (including *SKP1*, *WNT8A, CYP2E1*, and *NBPF9*) achieved the best performance in cross validation (Figure 5G; Figure S4G,H, Supporting Information), with an AUC of 0.911 (specificity = 87.1% and sensitivity = 87.8%). The test set also achieved a high AUC of 0.86 (AUC = 0.90 and AUC = 0.82 for Day 0 and Day 5 samples in the test set, respectively; Figure S4I, Supporting Information). The high accuracy in predicting Day 0 samples suggests that our 11‐gene 5hmC signature could serve as a promising pre‐treatment biomarker to refine patient selection of AZA‐HiDAC‐Mito therapy.

Given the ease of collection and less invasive properties, peripheral blood samples are generally considered as a preferred source for biomarker development in clinical applications. We thus evaluated the agreement between PB and BM samples in predicting treatment response. When performing unsupervised hierarchical clustering on pre‐treatment 5hmC samples based on the contributing genes in XGBoost model, we found that BM and PB samples from same patient exhibited high concordance (Figure S5A, Supporting Information). Most paired BM and PB samples clustered closely to each other, and successfully clustered into the responder group or non‐responder group. Moreover, as for the 11‐gene‐5hmC model, we observed comparable AUC values for PB and BM samples in the test set (AUC = 0.84 and 0.86, respectively; Figure S5B, Supporting Information). These results collectively support the feasibility of PB samples in predicting treatment response to AZA‐HiDAC‐Mito therapy using the established 11‐gene 5hmC signature.

## 3 Discussion

As the treatment of AML moves toward a subset specific approach with targeted agents and combination regimens, identifying patients who may respond better to an HMA‐based therapy remains an unmet need. Previous studies have analyzed DNA methylation profiling as a biomarker for response to HMA‐based treatment in patients with myelodysplastic syndrome or AML. However, these studies have produced mixed results and none have established an epigenetic predictor of responsiveness to HMAs.^[^ ^29^ ^]^ These studies focused on global methylation using long interspersed nuclear element (LINE) methylation, methylation patterns in specific tumor suppressor genes, and an aberrantly hypermethylated gene signature, but overall there was no reliable significant predictor of treatment response. Of note, these studies included other HMA combinations or HMA monotherapy for multiple cycles, whereas our study utilized AZA as epigenetic priming prior to cytotoxic therapy. In a phase 1 study of epigenetic priming with decitabine prior to cytotoxic therapy with cytarabine/daunorubicin in patients with AML, pre‐treatment and post‐priming DNA methylation levels of *CDKN2B*, *LINE1*, and *HISTH2AA* were not predictive of treatment response.^[^ ^30^ ^]^

While overall 5hmC levels are found to vary among patients with AML and show an inverse correlation with patient survival,^[^ ^31^ ^]^ the feasibility of using 5hmC profiles to predict the responsiveness to a particular treatment has yet to be assessed. To our knowledge, our work is the first effort to identify a 5hmC predictive biomarker for treatment response in AML. Although 5hmC profiling is investigational and not yet a part of the clinical pre‐treatment evaluation of patients with AML, it is a quick and sensitive method, which requires only a limited amount of genomic DNA.^[^ ^26^ ^]^ Using 5hmC profiling of peripheral blood and/or bone marrow biopsy samples in AML patients treated with AZA‐HiDAC‐Mito in a phase 1 clinical trial, we identified a pre‐treatment 11‐gene 5hmC signature as a predictive biomarker to identify patients who may benefit from AZA‐HiDAC‐Mito. Due to small sample size, we were not able to investigate the effect of cytogenetics and pathogenic mutations in the current model, and it would be important to incorporate these prognostic features along with the 5hmC signature in a larger prospective study.

In addition to biomarker identification, we also revealed mechanistic insights into the therapeutic response of AML patients to AZA‐HiDAC‐Mito. Among responders, we found an increased expression of genes involved in the cell cycle and DNA synthesis, suggesting that increased numbers of actively cycling cells may be associated with effective AZA‐HiDAC‐Mito response. In line with this, GMP‐like cells were speculated as a dominant proliferating malignant subset that was associated with treatment response. We further uncovered the in vivo effect of AZA treatment in inducing genes related to NK cell mediated cytotoxicity in responders. Clarifying whether this is a direct or indirect effect of AZA towards NK cells requires successor studies. Furthermore, analysis of patients receiving an AZA‐based regimen or non‐AZA‐based regimen revealed a unique role of NK cells in determining the response to AZA treatment. A combination of cellular fractions of GMP‐like cells and NK cells can better predict the treatment response to AZA‐HiDAC‐Mito therapy, suggesting a combined effect of tumor‐intrinsic state and immune microenvironment in governing the therapeutic response of AML patients.

## 4 Conclusions

Collectively, our findings show that cellular compositions are associated with treatment responses, and DNA 5hmC patterns in an 11‐gene signature can be used as a pre‐treatment biomarker for AZA‐HiDAC‐Mito therapy, which may help select patients who benefit from this regimen. The potential of this 5hmC gene signature in predicting treatment response merits validation in larger prospective trials as well as studies involving other novel HMA‐based combinations.

## 5 Experimental Section

### Study Subjects

Detailed phase 1 trial design methods for this study had been reported.^[^ ^6^ ^]^ The study population included patients age ≥ 18 years with high‐risk AML and Eastern Cooperative Oncology Group (ECOG) performance status 0–2. AML was defined by the 2008 criteria of the World Health Organization (WHO).^[^ ^32^ ^]^ Patients with high‐risk disease were included and defined as therapy related‐AML (t‐AML), relapsed/refractory AML (RR‐AML), de novo AML in patients age ≥ 60 years, AML arising from myelodysplastic syndrome (MDS‐AML), myeloproliferative neoplasms in blast phase (MPN‐BP), and chronic myelomonocytic myeloid leukemia (CMML‐AML). This single‐center trial was registered at www.clinicaltrials.gov as NCT01839240. All participants provided written informed consent.

### Trial Design

Cohorts of three patients were treated in a 3 + 3 dose escalation scheme. Patients received AZA at 37.5 mg m^−2^, 50 mg m^−2^, or 75 mg m^−2^ by subcutaneous administration (SC) or intravenous therapy (IV) once daily on Days 1–5 followed by cytarabine 3000 mg m^−2^ given IV over 4 h followed by mitoxantrone 30 mg m^−2^ given IV over 1 h once each on Day 6 and Day 10. The maximum dose of AZA to be explored was capped at 75 mg m^−2^. Cytarabine and mitoxantrone dose reductions were made for patients age ≥ 70 by 33% to 2000 mg m^−2^ of cytarabine and 20 mg m^−2^ of mitoxantrone. A research related bone marrow aspirate was performed pre‐treatment/prior to AZA administration (Day 0) and after AZA administration (Day 5). Mononuclear cells were extracted and samples were cryopreserved for future analysis. To evaluate the efficacy of this regimen, a nadir marrow biopsy was performed on Day 17 and a biopsy to assess remission status was done within 2 weeks of hematologic recovery (defined as absolute neutrophil count (ANC) ≥ 1000 per µL and platelet count ≥ 100 000 per µL), but no later than Day 42. Response criteria for complete remission (CR), CR with incomplete count recovery (CRi), and treatment failure (TF) were defined according to the 2010 ELN Working Group recommendations.^[^ ^33^ ^]^ The overall response rate was defined as CR + CRi, and these patients were defined as responders to treatment. Overall survival was defined as time from treatment to time of death. The data cutoff date was November 1, 2017. The clinical results of the trial had been published.^[^ ^6^ ^]^

### DNA and RNA Isolation

DNA was extracted from bone marrow or peripheral blood using the Gentra Puregene Cell kit (Qiagen, Valencia, CA) according to the manufacturer's directions. RNA was extracted using TRIzol Reagent (Thermo Fisher Scientific, Waltham, MA) according to the manufacturer's directions.

### RNA Sequencing

mRNA was extracted from 1 µg of total RNA by using Dynabeads mRNA Direct kit (Ambion). For each sample, 20 ng of mRNA was used for library construction by using TruSeq stranded mRNA sample preparation kit (Illumina). Libraries were sequenced on Illumina Hiseq 4000.

### Nano‐hmC‐Seal

Nano‐hmC‐Seal (5hmC‐seq) was performed on 120 bone marrow (BM) and peripheral blood (PB) samples from 40 patients collected at Day 0 and/or Day 5, as previously described with minor changes.^[^ ^26^ ^]^ Libraries were prepared with KAPA Hyperplus kit (KAPA KK8515) using extracted genomic DNA from patient BM or PB mononuclear cells. Briefly, 50 ng genomic DNA in 14 µL H_2_O was fragmented at 37 °C for 20 min by addition of 2 µL of 10x KAPA Fragmentation Buffer and 4 µL of KAPA Fragmentation Enzyme. The fragmented DNA was end‐polished at 65 °C for 30 min by adding 2.8 µL of End Repair & A‐Tailing Buffer and 1.2 µL of End Repair & A‐Tailing Enzyme Mix. Three microliters of 1.5 µm Adapter (Bioo Scientific NOVA‐514103) were added followed by 12 µL of Ligation Buffer and 4 µL DNA Ligase. The mixture was incubated at 20 °C for 1 h. Libraries were then purified by DNA Clean and Concentrator kit (Zymo D4013) and eluted in 20 µL H_2_O. *β*‐GT labeling was then performed by addition of 0.85 µL self‐synthesized 3 mm N_3_‐UDG and 2.5 µL of T4‐*β*GT (Thermo EO0831) at 37 °C for 2 h. Azide labeled DNA libraries were then purified by DNA Clean and Concentrator kit (Zymo D4013) and eluted in 30 µL H_2_O. Libraries were further biotinylated by addition of 1 µL 4.5 mm (Sigma 760 749) DBCO‐PEG_4_‐Biotin and incubated at 37 °C for 2 h. Biotinylated DNA libraries were then purified by DNA Clean and Concentrator kit (Zymo D4013) and eluted in 30 µL H_2_O. The biotinylated DNA was further enriched by 5 µL of M‐270 Streptavidin beads (Thermo 65 305) and incubated at room temperature for 30 min. The beads were washed 3 times with Wash Buffer (5 mm Tris‐HCl (pH 7.5); 0.5 mm EDTA; 1 m NaCl; 0.05% Tween 20) and resuspended in 20 µL H_2_O. Libraries were amplified with on‐bead PCR by addition of 5 µL Primer Mix (KAPA KK8515) and 25 µL of Enzyme Mix (KAPA KK8515) with following condition (98 ˚C 30 s; 98 ˚C 15 s; 60 ˚C 30 s; 72 ˚C 30 s; Repeat 14 cycles; 72 ˚C 1 min). Post‐amplification cleanup was performed by adding 0.9x Ampure beads (Beckman Coulter A63880); beads were washed twice with 80% ethanol and eluted in 50 µL H_2_O. Libraries were sequenced on Illumina NextSeq 500.

### RNA‐Seq Data Processing

The quality control for raw sequence data was performed by FASTQC version 0.11.8.^[^ ^34^ ^]^ The reads were then aligned to the UCSC hg19 reference genome by STAR‐2.5.3 software.^[^ ^35^ ^]^ Gene counts were analyzed by HOMER software.^[^ ^36^ ^]^ BM and PB samples collected at Day 0 from patients treated with AZA‐HiDAC‐Mito were used to perform differential analysis. Differentially expressed genes between responders and non‐responders were detected by DESeq2.^[^ ^37^ ^]^ The threshold of differentially expressed genes was set to p.adj ≤ 0.1 and |log_2_ FoldChange| ≥ 0.5. Clustering analysis was performed with pheatmap package version 1.0.12. Annotation and genome files (Homo sapiens UCSC hg19) were downloaded from iGenomes.

### Gene Module and Pathway Analysis

For gene module analysis, Pearson's correlation coefficients were first calculated between each pair of differentially expressed genes (DEGs) based on log_2_‐scaled normalized expression by variance stabilizing transformation (vst) method. A hierarchical clustering based on the Euclidean distance was then employed to separate genes into four modules. STRING database was utilized to extend gene modules by adding direct interacting genes that had a mean expression over 3 Transcripts Per Million (TPM).^[^ ^14^ ^]^ Functional enrichment analysis was next performed with Metascape for each module network.^[^ ^38^ ^]^ For GSEA analysis, clusterProfiler was utilized.^[^ ^39^ ^]^ Pathview package was used for visualization of transcriptional changes in indicated pathways. GSVA package was used for calculating gene‐set enrichment scores per sample with default settings except for “mx.diff = F”. For patients receiving AZA‐HiDAC‐Mito therapy, log_2_‐scaled TPM expression was used; for public Beat AML cohort, the normalized expression matrix from https://biodev.github.io/BeatAML2 was used. The LSC17 scores were calculated per sample as the sum of the log_2_‐transformed TPM values for the 17 genes weighted by the regression coefficients, as described previously.^[^ ^21a^ ^]^

### Digital Cytometry

Gene expression deconvolution was performed on CIBERSORTx web portal with default setting. In brief, reference signature matrix was built by CIBERSORTx based on gene expression of 13,653 cells belonging to six malignant subsets (HSC‐like, Prog‐like, GMP‐like, ProMono‐like, Mono‐like, cDC‐like), and 7 non‐leukemic immune populations, including Mature B cell (B), Conventional dendritic cell (cDC), Cytotoxic T Lymphocyte (CTL), Monocyte, Plasma cell (Plasma), Naïve T cell (T), and Natural Killer cell (NK). Bulk RNA‐seq data were normalized with TPM and then deconvoluted using S‐mode batch correction and relative mode. The inferred fractions were scaled to a sum of 1 for malignant subsets or immune populations, respectively.

### Survival Analysis

Kaplan‐Meier survival analysis was calculated with survival R package (version 3.1‐7) and visualized by the survminer R package (version 0.4.6). In multivariable analysis, age and sex features were incorporated into the Cox regression models. For Beat AML cohort, the available ELN2017 risk classification was also considered.

### 5hmC‐Seq Data Analysis

The quality control for raw sequence data was performed by FASTQC version 0.11.8.^[^ ^34^ ^]^ The 5hmC reads were then mapped to the UCSC hg19 reference genome by STAR‐2.5.3 software with parameter “–alignIntronMax 1 –alignEndsType EndToEnd”. The de‐duplication was performed using the parameter “‐tbp 5” in makeTagDirectory of HOMER software, and the gene counts matrix was generated by the HOMER's analyzeRepeats. DESeq2 was utilized to identify differentially hydroxy‐methylated genes (DhMGs) upon AZA treatment (Day 5 vs Day 0) under threshold *p* value <0.01 and |log_2_ FoldChange| ≥ 0.5.

### Machine Learning Based on 5hmC‐Seq Data

A total of 120 BM/PB samples were collected from 40 patients for 5hmC profiling. The first batch of sequenced samples consisted of 80 samples obtained from 22 patients (47 samples from 13 responders and 33 samples from 9 non‐responders), which were selected at random and without consideration of their treatment response status, to serve as the train set. The remaining 40 samples obtained from 18 patients were sequenced in the second batch and served as the test set (28 samples from 12 responders and 12 samples from 6 non‐responders). The responders were set as case observations (positive label), and non‐responders were set as control observations (negative label) for the machine learning algorithm. The detailed patient id and clinical information of both train set and test set can be found in Table S1 (Supporting Information). Rlog‐normalized 5hmC levels for each gene were used to build classifier by XGBoost with python API (version 3.6.6). Probabilities estimation was then generated by “predict_proba” method. The performance was evaluated by patient‐based five‐fold cross validation. The importance of each gene (F‐score) was calculated by “get_fscore” function. Top 11 genes with highest F score were selected to rebuild the classifier. To set up a negative control for the machine learning models, the response status of patients were randomly shuffled in the train set, and then the XGBoost models were trained on either all genes or just the top 11 contributing genes.

### Statistical Analyses

All statistical analyses were performed in R 3.6.0 software. The Pearson's correlation was used unless specified otherwise. For comparison of responders and non‐responders, two‐tailed unpaired Student's *t* tests or Wilcoxon rank sum tests were performed. For comparison of paired Day 0 and Day 5 samples, two‐tailed paired Student's *t* tests were used. For Kaplan‐Meier survival curves, the *p* values were calculated using two‐tailed log rank tests. Multivariable Cox models for overall survival were used to adjust for potential confounders including age and sex. Statistical significance was set at *p* < 0.05. All boxplots indicate median (center), 25th and 75th percentiles (boundaries of the box), and minimum and maximum (whiskers).

### Ethical Statement

This study was reviewed and approved by the institutional review board at the University of Chicago (IRB 12–0111).

## Conflict of Interest

C.H. is a scientific founder, a member of the scientific advisory board and equity holder of Aferna Bio, Inc. and AccuaDX Inc., a scientific cofounder and equity holder of Accent Therapeutics, Inc., and a member of the scientific advisory board of Rona Therapeutics; O.O has served on advisory boards convened by ABBVIE, Celgene/BMS, CTIBiopharma, Novartis, Impact Biomedicines; W.S. has served on advisory boards convened by Agios, Amgen, Astra Zeneca, Beam, Glaxo Smith Kline, Jazz, Kite, Kronos, Kura, Newave, Pfizer, Pluristem, Servier, Syndax.

## Author Contributions

G.L., L.W., Q.Y., and K.C. contributed equally to this work. O.O., C.H., and D.H. designed the study and supervised the research. Q.Y. and N.F. performed experiments. K.C., O.O., and W.S. collected samples. G.L., L.W., and C.C. performed data analysis. D.H., G.L., Q.Y., and C.H. wrote the manuscript with input from L.W., K.C., O.O., W.S., W. Z., and C.C. All authors discussed the results and commented on the manuscript.

## Supporting information

Supporting Information

Supporting Information

## Data Availability

The data that support the findings of this study are openly available in Gene expression Ominibus (GEO) at https://www.ncbi.nlm.nih.gov/geo, reference number 152431 and Genome Sequence Archieve (GSA) at https://ngdc.cncb.ac.cn/gsa‐human, reference number HRA000372.
