# Supplementary material for: Cellular Composition and 5hmC Signature Predict the Treatment Response of AML Patients to Azacitidine Combined with Chemotherapy
Source: Adv Sci (Weinh). 2023 Jun 4;10(23):2300445. doi: 10.1002/advs.202300445 (PMC10427370; doi:10.1002/advs.202300445)
Supplement: Supplementary file 1 — Supporting Information [file ADVS-10-2300445-s001.pdf]

ADVANCED SCIENCE
Open Access

# Supporting Information

for *Adv. Sci.*, DOI 10.1002/advs.202300445

Cellular Composition and 5hmC Signature Predict the Treatment Response of AML Patients to Azacitidine Combined with Chemotherapy

*Guanghao Liang, Linchen Wang, Qiancheng You, Kirk Cahill, Chuanyuan Chen, Wei Zhang, Noreen Fulton, Wendy Stock, Olatoyosi Odenike*, Chuan He* and Dali Han**

# Supporting Information

**Cellular Composition and 5hmC Signature Predict the Treatment Response of AML Patients to Azacitidine Combined with Chemotherapy**

*Guanghao Liang, Linchen Wang, Qiancheng You, Kirk E. Cahill, Chuanyuan Chen, Wei Zhang, Noreen Fulton, Wendy Stock, Olatoyosi Odenike*, Chuan He*, Dali Han**

*G. Liang, L. Wang, Q. You, and K. Cahill contributed equally to this work.*

## 1. Supporting Tables

**Table S1. Clinical characteristics and sample information for patients treated with AZA-HiDAC-Mito therapy** (see Excel file).

**Table S2. Univariate and multivariable Cox regression models for overall survival of patients receiving Azacitidine treatment in Beat AML cohort.**

| Comparison | Univariate hazard ratio (95% CI); p value | Multivariate hazard ratio (95% CI); p value |
|---|---|---|
| high vs low (NK cell abundance) | 0.40 (0.19-0.83); p=0.015 | 0.32 (0.14-0.73); p=0.0070 |
| high vs low (NK cell signature) | 0.37 (0.18-0.76); p=0.0073 | 0.18 (0.070-0.44); p=0.00022 |

**Table S3. Univariate and multivariable Cox regression models for overall survival of patients in AZA-HiDAC-Mito cohort.**

| Comparison | Univariate hazard ratio (95% CI); p value | Multivariate hazard ratio (95% CI); p value |
|---|---|---|
| high vs low (DhMG number) | 0.22 (0.056-0.84); p=0.026 | 0.12 (0.023-0.59); p=0.0098 |
| low vs high (5hmC correlation) | 0.16 (0.050-0.52); p=0.0024 | 0.066 (0.015-0.29); p=0.00031 |

## 2. Supporting Figures

Figure S1

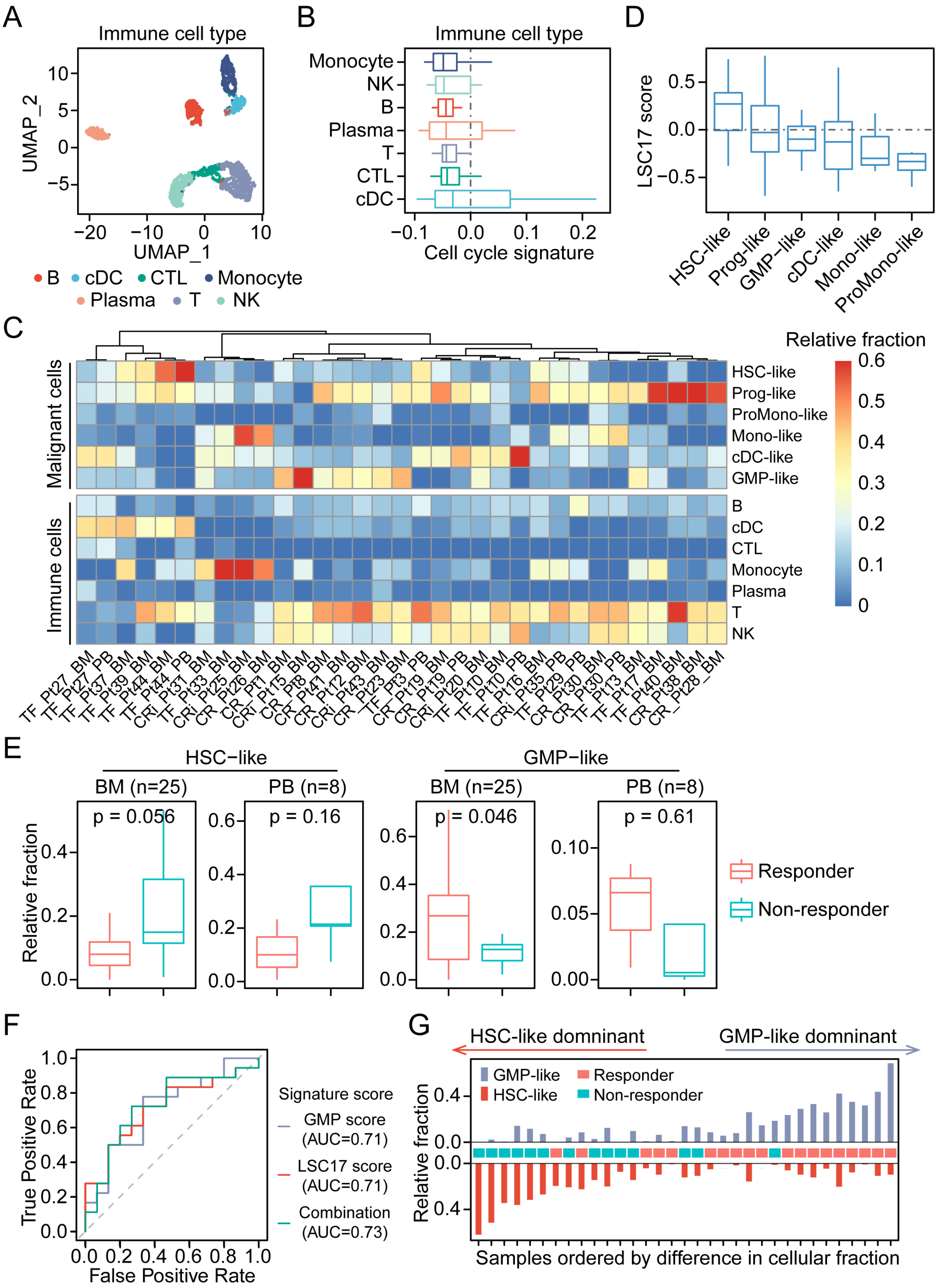


**Figure S1. Cellular compositions of patients receiving AZA-HiDAC-Mito therapy**

A) UMAP visualization of immune subsets from the public single-cell transcriptome (van Galen et al). Seven immune cell types were analyzed, including Mature B cell (B), Conventional dendritic cell (cDC), Cytotoxic T Lymphocyte (CTL), Monocyte,

Plasma cell (Plasma), Naïve T cell (T), and Natural Killer cell (NK).

B) Boxplot showing the aggregated gene expression of cell cycle signature in each immune subset per patients in the scRNA-seq dataset.

C) Heatmap showing the deconvolution result for malignant cells and immune cells. The estimated fractions were scaled to a sum of 1 for malignant subsets or immune populations, respectively.

D) Boxplot showing the pseudo-bulk result of LSC17 score in each malignant subset per patients in the scRNA-seq dataset.

E) Boxplot showing the estimated relative abundance of HSC-like cells (left) and GMP-like cells (right) within malignant subsets. Samples from BM and PB were analyzed separately. p values were calculated with two-sided Students t -test.

F) Receiving operating curve (ROC) analysis: Using score of GMP-like signature, LSC17 signature, or their combination (difference between the two signature scores) to predict responders.

G) Relative fractions of GMP-like subset and HSC-like subset among malignant cells for each pre-treatment samples.

Figure S2

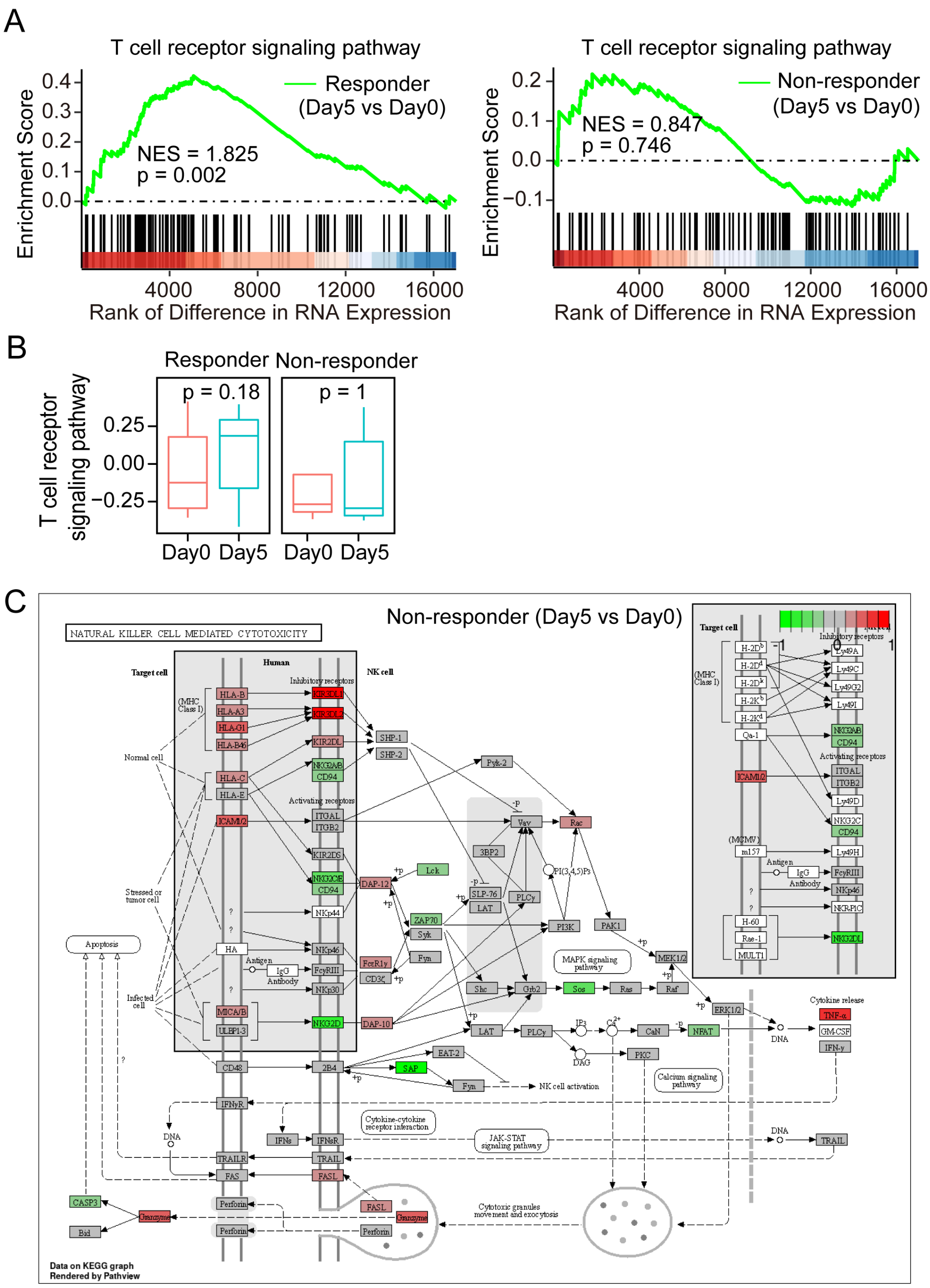


**Figure S2. Transcriptional changes upon AZA treatment**

A) GSEA to assess the enrichment of T cell receptor signaling pathway upon AZA treatment for 5 days in responders (left) and non-responders (right). NES, normalized enrichment score; p value was calculated with permutation test.

B) Boxplot showing GSVA scores of T cell receptor signaling pathway. Paired samples were used in comparison of Day 5 and Day 0. p values were calculated with

two-sided paired Students t -test.

C) Pathview map showing the gene expression changes between Day 5 and Day 0 in non-responders in Natural killer cell mediated cytotoxicity pathway. The mapped color indicates $log_2$(fold change) of Day 5 vs Day 0.

## Figure S3

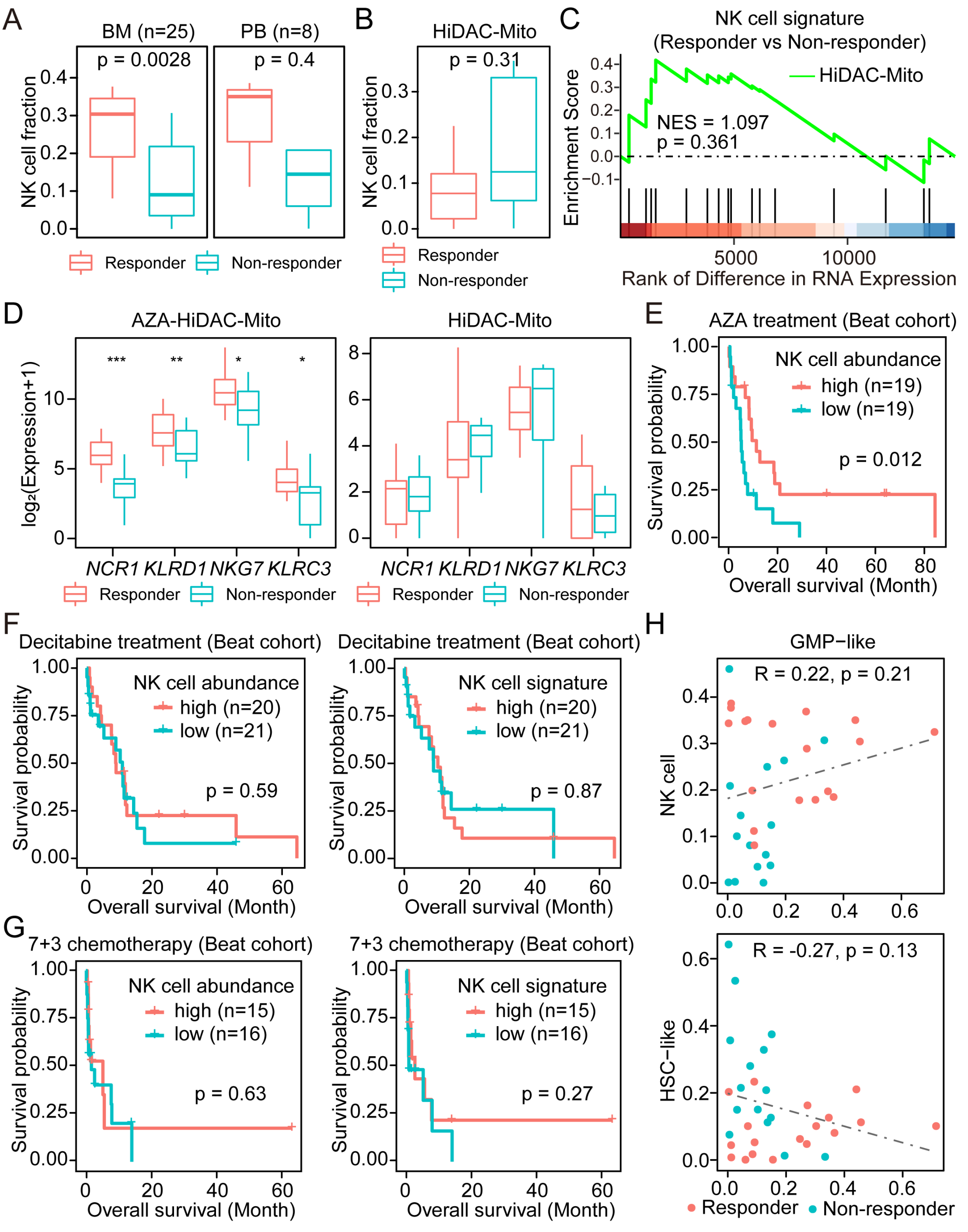


**Figure S3. Enrichment of NK cells predicted favorable clinical outcomes in AZA-based therapies**

A) Boxplot showing the estimated relative abundance of NK cells within immune subsets. Samples from BM and PB were analyzed separately. p values were calculated with two-sided Students t -test.

B) Boxplot showing the estimated relative abundance of NK cells within immune cells in patients receiving HiDAC-Mito only therapy. p values were calculated with two-sided Students t -test. Fractions of BM/PB samples from same patients were averaged.

C) GSEA to assess the enrichment of a curated NK cell signature in responders of HiDAC-Mito only therapy, comparing to non-responders. NES, normalized enrichment score; p value was calculated with permutation test.

D) Boxplot showing the expression levels of NK cell marker genes in patients receiving AZA-HiDAC-Mito therapy and HiDAC-Mito therapy, respectively. p values were calculated with Wald test by DESeq2. *p <0.05; **p <0.01; ***p <0.001.

E) Kaplan-Meier curve of overall survival for patients receiving AZA treatment in Beat AML cohort. Patients were equally divided into two groups based on estimated NK cell abundance by CIBERSORTx. p value was calculated with a two-tailed log rank test.

F-G) Kaplan-Meier curve of overall survival for patients receiving Decitabine treatment (F) or '7+3' chemotherapy (G) in Beat AML cohort. Patients were equally divided into two groups based on estimated NK cell abundance (left) or aggregated expression of a NK cell signature (right). p value was calculated with a two-tailed log rank test.

H) Correlation of fractions between GMP-like cells and NK cells (top) or HSC-like cells (bottom) in Day 0 samples.

Figure S4

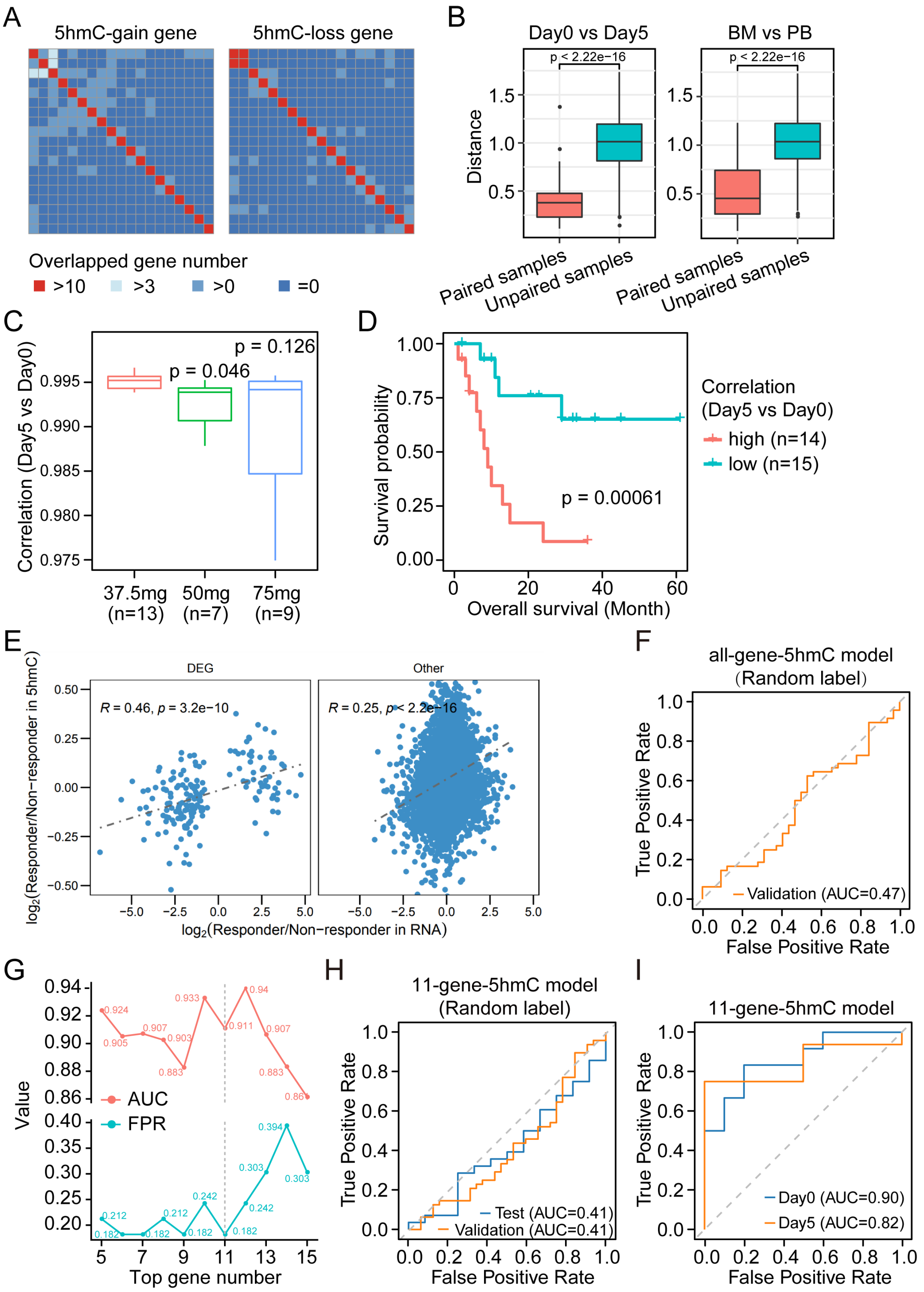


**Figure S4. 5hmC profiling for patients receiving AZA-HiDAC-Mito therapy**

A) Heatmap showing the overlap of 5hmC-gain genes (left) and 5hmC-loss genes (right) upon AZA treatment for 19 patients with both BM and PB samples at Day 0 and Day 5 (4 samples for each patient).

B) Pearson's distance of 5hmC samples from the same patients compared to distance

of samples from different patients. p value was calculated with a Wilcoxon rank sum test.

C) Boxplot showing Spearman's correlation between Day 0 and Day 5 paired samples from BM. p value was calculated with a Wilcoxon rank sum test.

D) Kaplan-Meier survival curve of 29 patients with paired Day 0 and Day 5 samples from BM. Patients were divided into two groups based on the median of pairwise Spearman's correlation between Day 0 and Day 5 BM samples. p value was calculated with a two-tailed log rank test.

E) Comparison of $\log_2$(fold change) between responders and non-responders in RNA expression and 5hmC levels. Pearson's correlations were shown.

F) ROC curves for the performance of XGBoost classifiers trained with all genes. The response status of patients in the train set were randomly shuffled.

G) The performance of classifiers trained with varying numbers of top 5hmC features. The red line indicates the AUCs calculated by patient-based 5-fold cross validation. The cyan line indicates false positive rates (FPR).

H) ROC curves for the performance of the XGBoost classifiers trained with top 11 contributing genes. The response status of patients in the train set were randomly shuffled.

I) ROC curve for the performance of the XGBoost classifier based on the 11-gene-5hmC signature in the test set. Day 0 and Day 5 samples were evaluated separately.

Figure S5

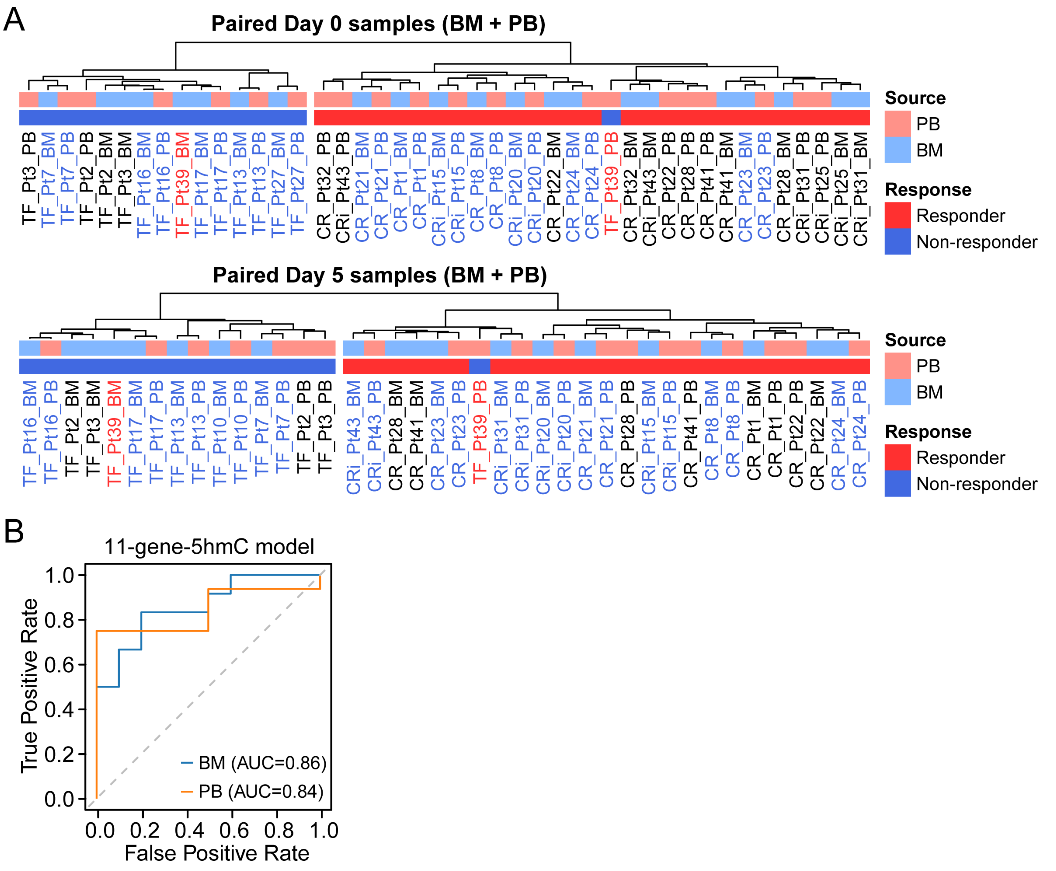


**Figure S5. BM and PB samples exhibited concordance in distinguishing responders and non-responders**

A) Heatmap showing hierarchical clustering of paired BM and PB samples collected at Day 0 (top) or Day 5 (bottom) based on the 5hmC levels of contributing genes from XGBoost model. Samples from same patient were colored in blue if they clustered most closely.

B) ROC curves for the performance of the XGBoost classifier based on the 11-gene-5hmC signature in the test set. BM and PB samples were evaluated separately.
